# Effectiveness and Acceptability of e- and m-Health Interventions to Promote Physical Activity and Prevent Falls in Nursing Homes—A Systematic Review

**DOI:** 10.3389/fphys.2022.894397

**Published:** 2022-05-20

**Authors:** Jonathan Diener, Sabine Rayling, Jelena Bezold, Janina Krell-Roesch, Alexander Woll, Kathrin Wunsch

**Affiliations:** Institute of Sports and Sports Science, Karlsruhe Institute of Technology, Karlsruhe, Germany

**Keywords:** physical activity, fall prevention, e-health, m-health, exergaming, nursing home, long-term care, systematic review

## Abstract

Age-related decreases in physical activity (PA) and a decline in physical functioning lead to increased fall risk. As falls are a major cause of accidental deaths and hospitalization in older adults, PA promotion and fall prevention are important measures, especially in nursing homes (NH). With advances in information and communication technology, e- and m-health solutions have been developed to positively influence various health-related factors. To date, only little research exists on the implementation of these technologies to promote health in NH. Therefore, the objective of this systematic review was to provide an overview of the effectiveness, acceptability, and feasibility of e- and m-health interventions aimed at promoting PA and preventing falls in NH. Additionally, the effectiveness of such interventions regarding the secondary outcomes physical function, cognitive function, neuropsychiatric symptoms, and psychosocial status was examined. A systematic literature search was performed in five databases and studies published until 15 November 2021, were considered for inclusion. All studies that examined the effectiveness and/or the acceptability and feasibility of e- or m-health interventions in promoting PA and preventing falls in NH, without restriction on language or date of publication, were included in the final synthesis. Of the 1,358 records retrieved, 28 studies were included in this systematic review. Twenty-four studies contained digital exergaming as an intervention or as a part of the intervention, the four additional studies on e-health interventions only examined a small number of outcomes. No m-health intervention study was identified. Data synthesis indicates that exergaming may be effective in reducing the number of falls and fall risk in NH residents. Several significant improvements were also reported regarding secondary outcomes albeit not consistent across studies. No conclusion can be drawn about the effects of exergaming and other e-health interventions on PA, as data is scarce. E-health interventions were mostly reported as feasible and well accepted by NH residents. However, these findings may not be applicable to NH residents with advanced physical and/or cognitive impairments, since they were excluded in many studies. Therefore, more research examining other digital solutions besides exergaming to promote PA in this specific population is critical.

**Systematic Review Registration:** https://www.crd.york.ac.uk/prospero/, identifier CRD42021289488

## 1 Introduction

The global increase in chronic diseases due to an aging population represents a major challenge for health care (Prince et al., 2015). With increasing age, impairments in physical performance (e.g., changes in gait, balance problems) and difficulties with activities of daily living are likely to occur (Colón-Emeric et al., 2013; Osoba et al., 2019). These impairments often lead to a reduction in physical activity (PA), which in turn leads to further deconditioning (Chen, 2010). Especially among nursing home (NH) residents, physical inactivity is a widespread health risk (Grönstedt et al., 2011) and is associated with higher frailty (Buckinx et al., 2017). Overall, NH residents are less physically active than older people living independently at home (Egerton and Brauer, 2009).

The age-related decline in PA and associated deterioration in physical functioning are also related to fall risk (Ylitalo et al., 2021) which represents a major issue in NH; NH residents have fall rates that are almost three times higher than community-dwelling older adults. Falls in NH are also more likely to result in serious complications, as 10–25% of falls result in fractures or injuries (Rubenstein, 2006). Two large-scale studies in German NH found that 4.6% of NH residents suffered from a fall within the 14-days observation period (Lahmann et al., 2014), and the rates range between 2.18 (men) and 1.49 (women) falls per person-year (Rapp et al., 2012).

Falls can lead to fear of falling (Denkinger et al., 2015) and thus to decreased falls efficacy, which refers to the confidence in being able to perform a variety of activities of daily living without a fall (Yardley et al., 2005). In addition, fear of falling often leads to a reduction in PA (Jefferis et al., 2014; Kader et al., 2016), which is related to social isolation and may contribute to increased health risks associated with isolation (Schrempft et al., 2019) such as depression or a reduced quality of life (Nicholson, 2012). Additionally, neuropsychiatric symptoms such as anxiety and depression seem to be conditions that predispose to falls (Dionyssiotis, 2012; Pasquetti et al., 2014). Similarly, cognitive deterioration also appears to be associated with inactivity and fall risk. There is evidence that NH residents with severe cognitive impairment are less physically active than residents with mild cognitive impairment (Eggermont and Scherder, 2008; Grönstedt et al., 2011), and that a decline in cognitive function likely contributes to an increased risk of falls (Allali et al., 2008; Lamoth et al., 2011).

Research has shown that long-term exercise and balance training can prevent falls in NH residents (Wang and Tian, 2022). Conflicting results have been reported regarding the impact of exercise on cognitive function in NH residents. While Cordes et al. (2021a) and Thurm et al. (2011) reported positive results, Henskens et al. (2018) and Toots et al. (2017) found no effects. In terms of physical functioning, exercise interventions in NH commonly reveal significant positive effects (Barrett et al., 2021; Cordes et al., 2021b). Additionally, PA interventions have been shown to positively impact neuropsychiatric symptoms in older adults such as depression (Bridle et al., 2012) or anxiety symptoms (Mochcovitch et al., 2016), as well as on psychosocial status including quality of life (Bauman et al., 2016) and fear of falling (Kumar et al., 2014).

With advances in information and communication technology, innovative e- and m-health solutions have been developed to positively influence PA and to reduce accidental falls. According to Eng (2001, *p*. 1), e-Health was defined as “the use of emerging information and communication technology, especially the Internet, to improve or enable health and healthcare.” A subdivision of e-Health, m-health, was defined as “medical and public health practice supported by mobile devices, such as mobile phones, patient monitoring devices, personal digital assistants (PDAs), and other wireless devices” (World Health Organisation, 2011, *p*. 6). E- and m-health technologies offer new opportunities for a more person-centered care that more effectively considers individual abilities and impairments (Goh et al., 2017). Given the high heterogeneity of the NH population in terms of their cognitive and physical functioning, it is difficult to adjust conventional intervention programs (i.e., that are not based on e- or m-health) to individual needs (Trautwein et al., 2020; Bezold, Trautwein et al., 2021; Barisch-Fritz et al., 2022a). Furthermore, the interactive nature and implementation of game elements in digital applications can provide an enjoyable experience for elderly users (Syed-Abdul et al., 2019).

For the successful implementation of digital health interventions designed for older adults, the feasibility regarding organizational and systems readiness as well as the acceptability of the digital intervention are particularly important. In general, digital solutions are more likely to be accepted by healthcare professionals and patients if they show high usability and offer the ability to adapt to the population of interest as well as to individual preferences and needs (Matthew-Maich et al., 2016; Spann and Stewart, 2018). Usability is the capability of a system or device to be used by humans easily and effectively (Shackel, 2009). No matter how advanced and innovative a technology may be, if it proves unfeasible or unaccepted in practice, it is of little or no value (Harte et al., 2017).

Given the aforementioned benefits of digital health interventions, it is surprising that only few digital solutions to promote the health of NH residents have been developed and little research on the usability and feasibility exists (Barisch-Fritz et al., 2020). In addition, information on which e- and m-health technologies are predominantly used, and which features these interventions contain, is limited. Accordingly, to date, little is known about the effectiveness of these interventions in NH residents. Therefore, the aim of this systematic review was to provide an overview of the effectiveness, acceptability, and feasibility of e- and m-health interventions in promoting PA and preventing falls in NH. Secondary outcomes include the effectiveness of the interventions regarding physical and cognitive function, neuropsychiatric symptoms, and psychosocial status of NH residents.

## 2 Methods

This review is structured according to the updated version of the Preferred Reporting Items for Systematic Reviews and Meta-Analysis (PRISMA) statement (Page et al., 2021). It was submitted to the international prospective register of systematic reviews PROSPERO on 17 November 2021, and registered on 18 December 2021 (ID: CRD42021289488).

### 2.1 Eligibility Criteria

Population and study design: Randomized or non-randomized intervention trials that focused exclusively on NH residents were included. Studies only among individuals with a specific health condition (e.g., only individuals with dementia) or studies that (partly) included community-dwelling older adults living at home or in assisted living facilities were excluded. The rationale for the exclusion of studies involving only individuals with a specific health condition was to facilitate comparison of the studies and to be able to provide recommendations for the broader nursing home population.

Intervention: All interventions were included if they involved PA and/or PA promotion strategies (e.g., counselling, reminders) and/or multimodal fall prevention strategies (e.g., education, environmental modifications, and exercise). Moreover, trials had to include an e- or m-health element (e.g., mobile applications, video games, conversational agents) as the primary or a major intervention delivery mode. Intervention studies where only the assessment of PA or falls was performed using e- or m-Health technologies as well as robot-assisted interventions were excluded.

Outcomes: Included studies had to report on at least one of the primary outcomes as follows: PA (e.g., steps per day, PA levels), accidental falls (e.g., fall rates, fall risk (sensor-based evaluation or clinical assessment through performance-oriented balance tests such as Timed Up and Go Test or Berg Balance Scale)), feasibility (e.g., adherence), and/or NH residents’ or staff’s acceptability (e.g., usability, attitude toward the use, perceived enjoyment) of the e- or m-health intervention.

### 2.2 Information Sources

For the identification of relevant studies, the databases PubMed, Scopus, SPORTDiscuss and Web of Science Core Collection were searched until 15 November 2021.

Subsequently, the reference lists of the studies included in this systematic review as well as of previously published systematic reviews on a similar topic were screened for further relevant publications (a list of reviews screened can be found in the Supplementary Material). For both backward and forward citation searching, Web of Science [Science Citation Index] and Scopus were used. The first round of citation searching was completed on 5 December 2021.

Afterwards, the first 200 results from a Google Scholar search were additionally screened on 7 December 2021, as recommended by Bramer et al. (2017).

Lastly, the reference lists of studies included in this systematic review after the initial search in the four databases were scanned for further relevant publications. The identification of relevant studies was completed on 10 December 2021.

### 2.3 Search Strategy

The structure of the search strategy was based on the three main concepts examined in this review: NH residents, PA and fall prevention, as well as e- and m-Health. Both, free-text and MeSH terms were used, with a wide range of synonyms, related terms, and alternative spellings. The search terms were determined through group discussion within the research team. The final strategies were peer reviewed by a librarian, which involved proofreading the syntax, spelling and overall structure. Only articles published in scientific journals were considered, and no restrictions were applied in terms of publication date or language. To identify studies that included the use of e- and m-health, search terms such as virtual reality, wearable, cell phone [MeSH], smartphone, conversational agent and internet-based were used. Search terms used to find studies that focused on PA and fall prevention included physical activity, exercise [MeSH], mobility, training, gait [MeSH] and fall. Terms such as nursing homes [MeSH], long-term care, institutionalized and aged care facility were used to identify studies that targeted NH residents. The exact search strategy for each database utilized is provided in the Supplementary Table S2.

### 2.4 Selection Process

Two researchers (JD, SR) independently screened titles and abstracts of the articles retrieved. In case of disagreement, consensus was reached through discussion on which articles should be included in the full-text screening. If required, a third researcher (KW) was consulted to reach the final consensus. The same procedure was applied within the full-text screening. One article of which only the abstract was available in English was translated from Mandarin using the software DeepL Translator (Version 3.3; DeepL SE, 2022).

### 2.5 Data Collection Process and Data Items

Data were extracted into a data extraction form using a Microsoft Excel spreadsheet. Relevant data were extracted by one author (JD) and then verified by a second author (SR). Disagreements were solved as previously described. Data extracted from articles included general study information (e.g., authors, year), description of the study sample (e.g., size, age), description of the intervention (e.g., content of the intervention, duration), control description, measurement instruments as well as results of primary (PA, falls/fall risk, acceptability/feasibility) and secondary (cognitive and physical function, neuropsychiatric symptoms and psychosocial status) outcomes.

### 2.6 Study Risk of Bias Assessment

To determine the quality of the included studies, the assessment tool for quantitative studies of the Effective Public Health Practice Project (EPHPP) was used (Thomas et al., 2004). Each study was independently rated by two authors (JR, SR). In case of discrepancies regarding the assessment, a joint decision was reached during discussion. The EPHPP tool addresses five specific domains: selection bias, study design, blinding, confounders, validity, and reliability of data collection methods, as well as withdrawals and drop-outs. Based on the assessment process, studies were assigned an overall study quality (strong, moderate, or weak). If a given study was not rated “weak” in any of the subcategories, it was overall rated “strong”; if it was rated “weak” in one of the subcategories, it was overall rated “moderate”; and if it was rated “weak” in two or more of the subcategories, it was overall rated “weak”.

### 2.7 Synthesis Methods

Given the heterogeneity of the included studies in terms of intervention characteristics, study duration, and outcome measures, it was not feasible to perform a meta-analysis. Therefore, the data obtained were summarized narratively. Narrative synthesis of included studies was carried out following Popay et al. (2006). Sub-categories of the primary and secondary outcomes of interest were identified. Additionally, differences and similarities across significant and nonsignificant findings were analyzed in the context of intervention and population characteristics, as well as other study characteristics.

## 3 Results

### 3.1 Study Selection

**FIGURE 1:**
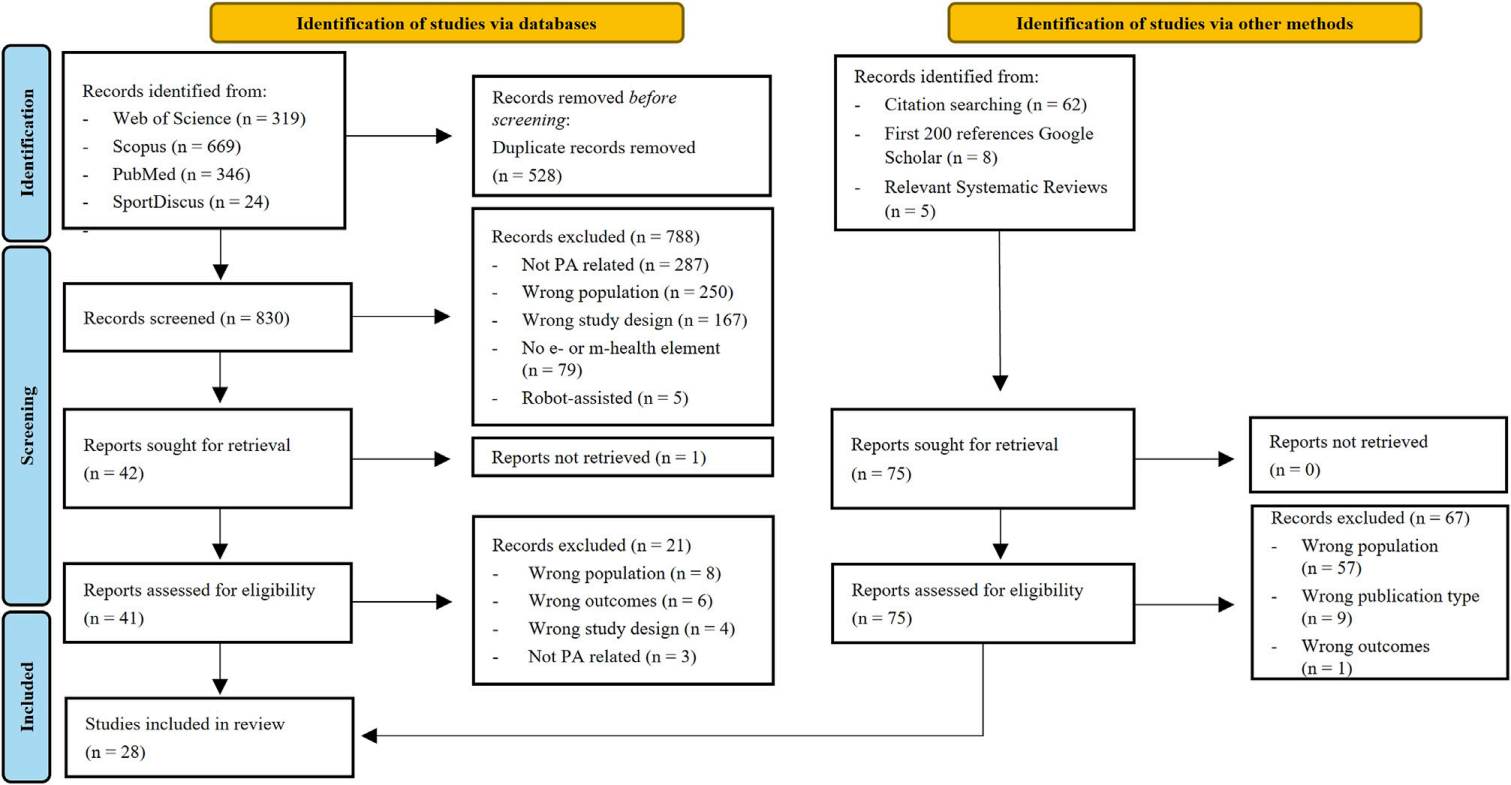
PRISMA flow diagram of the study selection process.

The database search retrieved 1,358 articles. After removing duplicates, title and abstracts of 830 articles were screened. Of these, 42 articles were reviewed in the full-text screening, resulting in the final inclusion of 20 articles. From the additional search, another 75 articles were included in the full-text screening, of which 8 met the inclusion criteria. Thus, a total of 28 articles were included in this systematic review (Chan et al., 2001; Brandt and Paniagua, 2011; Keogh et al., 2012; Pichierri et al., 2012; Ulbrecht et al., 2012; Janssen et al., 2013; Keogh et al., 2014; Fu et al., 2015; Grigorova-Petrova et al., 2015; Rogan et al., 2016; Yesilyaprak et al., 2016; Delbroek et al., 2017; Monteiro-Junior et al., 2017; Hutchinson et al., 2018; Kamińska et al., 2018; Sun et al., 2018; Taylor et al., 2018; Wu et al., 2019; de Bruin et al., 2020; Cicek et al., 2020; Fakhro et al., 2020; Rica et al., 2020; Babadi and Daneshmandi, 2021; Huang et al., 2021; Loggia et al., 2021; Marinus et al., 2021; Ramnath et al., 2021; Zahedian-Nasab et al., 2021). The PRISMA flow diagram of the study selection process is shown in Figure 1.

### 3.2 Risk of Bias in Studies

Ten studies were overall rated as “weak”, eight as “moderate” and 10 as “strong”. The lowest risk of bias was found in the domains “study design” and “dropouts and withdrawals”. In the domains “selection bias” and “blinding”, the vast majority was rated “moderate”. The “data collection” domain had the most “weak” ratings (9), however, at the same time many studies were rated as “strong” (19). The “confounders” domain was not applicable in 10 studies, while the quality of the remaining studies was mostly rated “strong” (16). The detailed results of the quality assessment are shown in Table 1.

**TABLE 1:** Results of methodological quality assessment.

Study	Selection bias	Study Design	Con-Founders	Blinding	Data Collection Methods	Withdrawals & drop-outs	Global rating
Babadi and Daneshmandi, (2021)	Moderate	Strong	Strong	Moderate	Strong	Strong	Strong
Brandt and Paniagua, (2011)	Weak	Weak	Weak	Weak	Weak	Strong	Weak
Chan et al. (2001)	Moderate	Weak	Weak	Moderate	Weak	Moderate	Weak
Cicek et al. (2020)	Strong	Strong	Strong	Weak	Strong	Moderate	Moderate
de Bruin et al. (2020)	Moderate	Strong	Strong	Strong	Strong	Strong	Strong
Delbroek et al. (2017)	Moderate	Strong	Strong	Moderate	Strong	Strong	Strong
Fakhro et al. (2020)	Strong	Strong	Strong	Moderate	Strong	Strong	Strong
Fu et al. (2015)	Moderate	Strong	Strong	Moderate	Strong	Strong	Strong
Grigorova-Petrova et al. (2015)	Weak	Moderate	Weak	Moderate	Strong	Weak	Weak
Huang et al. (2021)	Moderate	Weak	Weak	Moderate	Strong	Moderate	Weak
Hutchinson et al. (2018)	Weak	Weak	Weak	Moderate	Weak	Strong	Weak
Janssen et al. (2013)	Moderate	Strong	Moderate	Weak	Weak	Moderate	Weak
Kamińska et al. (2018)	Moderate	Moderate	Weak	Moderate	Weak	Strong	Weak
Keogh et al. (2012)	Moderate	Moderate	Weak	Moderate	Strong	Strong	Moderate
Keogh et al. (2014)	Moderate	Strong	Strong	Weak	Strong	Moderate	Moderate
Loggia et al. (2021)	Moderate	Strong	Strong	Moderate	Strong	Strong	Strong
Marinus et al. (2021)	Weak	Strong	Strong	Moderate	Strong	Strong	Moderate
Monteiro-Junior et al. (2017)	Moderate	Strong	Strong	Moderate	Strong	Moderate	Strong
Pichierri et al. (2012)	Strong	Strong	Strong	Weak	Weak	Weak	Weak
Ramnath et al. (2021)	Strong	Strong	Moderate	Moderate	Weak	Strong	Moderate
Rica et al. (2020)	Weak	Strong	Weak	Moderate	Strong	Weak	Weak
Rogan et al. (2016)	Moderate	Strong	Strong	Moderate	Strong	Strong	Strong
Sun et al. (2018)	Moderate	Strong	Strong	Moderate	Weak	Strong	Moderate
Taylor et al. (2018)	Moderate	Strong	Strong	Moderate	Strong	Strong	Strong
Ulbrecht et al. (2012)	Moderate	Moderate	Weak	Moderate	Weak	Weak	Weak
Wu et al. (2019)	Weak	Strong	Strong	Moderate	Strong	Moderate	Moderate
Yesilyaprak et al. (2016)	Weak	Strong	Strong	Moderate	Strong	Moderate	Moderate
Zahedian-Nasab et al. (2021)	Moderate	Strong	Strong	Moderate	Strong	Strong	Strong

### 3.3 Study Characteristics

Of the included studies, 10 were conducted in Europe (Belgium, Bulgaria, France, Germany, Netherlands, Poland, and Switzerland), 10 in Asia (China, Hongkong, Iran, Lebanon, Taiwan and Turkey), four in Australia/New Zealand, two in South America (Brazil) and one each in Africa (South Africa) and North America (United States ). All studies were published between 2001 and 2021, with most studies 21) being published since 2015.

Twelve studies were randomized controlled trials (RCT), two were cross-over RCTs, two were cluster-RCTs and four were controlled trials (CT). We also included four pre/post intervention studies and one cross-sectional explanatory study, one non-controlled, non-randomized pilot study, one feasibility study and one non-randomized, one-arm intervention trial. Two studies were labelled as letters to the editor (Brandt and Paniagua, 2011; Monteiro-Junior et al., 2017) but reported results of original research and were therefore included.

Twenty-four studies contained digital exergaming, with 17 studies using commercially available video gaming consoles (nine studies using the Nintendo Wii and eight studies the X-Box with Kinect sensor). Four studies used exergaming devices and software specifically designed for application in therapy and rehabilitation. In the remaining three studies, the open-source dance video game “StepMania” was employed, modified according to the participants preferences and needs (e.g., information on the screen was reduced to a minimum, music was selected according to the participants’ tastes). Two studies involved cybercycling (i.e., cycling on an ergometer in front of a screen on which the scenery changes based on the pedaling), one a 3D virtual reality-based horticultural therapy program using a virtual reality headset and one provided nursing services, including a falls prevention program, via videoconferencing.

The mean age of the subjects ranged from 66.9 to 88.9 years, while two studies did not report the age (Brandt and Paniagua, 2011; Hutchinson et al., 2018). Detailed study characteristics can be retrieved from Table 2 (general information, subject characteristics) and Table 3 (intervention and control characteristics).

**TABLE 2:** General information, study design, subject characteristics and setting of included studies.

Authors, year/Country	Study Design	Sampling (Groups, n, Gender, Age)	Inclusion Criteria	Exclusion Criteria	Setting
Babadi and Daneshmandi, (2021)/Iran	RCT	IG1: *n* = 12 (6f), 66.5 ± 3.8	No orthopedic disabilities or acute illnesses; age range between 60 and 75 years; able to stand independently for 90 s; no history of lower extremity fractures in the past 2 years	Impaired cognitive status (MMSE <24); serious visual or hearing impairment; more than three sessions missed during implementation of the training programs; neurological diseases	Nursing homes
		IG2: *n* = 12 (6f), 67.5 ± 3.1			
		CG: *n* = 12 (7f), 66.75 ± 3.27			
Brandt and Paniagua, (2011)/United States	non-controlled, non-randomized pilot study	*n* = 11	Not reported	Not reported	Long-term care facility
Chan et al. (2001)/Hongkong	non-randomized, one-arm, intervention trial	*n* = 198, 82 (range: 60–101)	Abbreviated Mental Test score of 6 or more; ability to communicate and to recall using the telenursing service, no diagnosis of dementia	Not reported	Residential nursing home
Cicek et al. (2020)/Turkey	CT	IG1: *n* = 16 (9f), 72.3 ± 6.0	Aged 65–85 years, MMSE score >20; sufficient communication skills; ability to walk 10 m with or without an assistive device	Serious neurological or orthopedic problems; diagnosed with psychiatric illness; moderate to severe cognitive impairment; advanced vision and hearing problems	Generic nursing home and rehabilitation center
		IG2: *n* = 14 (8f), 75.1 ± 5.5			
		CG: *n* = 14 (5f), 73.9 ± 4.6			
de Bruin et al. (2020)/Switzerland	RCT (sham control)	IG: *n* = 9 (5f), 86.1 ± 5.9	Aged 65 years or older; care-dependent: permanently depending on assistance or support in everyday activities; ability to stand for 1min with or without aids; minimum of 16 points in the MMSE; 6 or less points in the SPPB	Severe visual problems, acute fractures or thrombosis, epilepsy, migraine headaches, acute back pain, or active arthritis and amputation of the lower limb	Nursing home
		CG: *n* = 8 (5f), 90.3 ± 5.9			
Delbroek et al. (2017)/Belgium	RCT	IG: n = 10 (8f), 86.9 ± 5.6	Ability to walk 10 m repeatedly with walking aid; lived for at least 3 months at the residential care center; mild cognitive impairment (MoCa<26)	Participants who were still rehabilitating from a hospitalization; diagnosis of dementia; major sensory or motor impairments of the upper or lower extremities	Residential care center
		CG: n = 10 (5f), 87.5 ± 6.6			
Fakhro et al. (2020)/Lebanon	RCT	IG: *n* = 30	Age ≥65 years; impaired dynamic balance characterized by a TUG test score of >14 s; MoCA test score ≥23; good vision; institutionalized; vestibular disorders	Requirement for a wheelchair or walker for mobility; history of a fracture or orthopedic surgery to the lower extremities within the last 6 months; history of limb amputation	Geriatric centers; “institutionalized older adults”
		CG: *n* = 30			
		72.2 ± 5.2			
Fu et al. (2015)/Hongkong	RCT	IG: n = 30 (20f), 82.4 ± 3.8	Age ≥65 years; FAC grade 2 or 3; being alert and medically stable and able to follow instructions; history of falls in the previous year	Visual problems that might affect training; unable to follow instructions; history of seizure, stroke, parkinsonism; uncontrolled cardiovascular disease	Nursing home
		CG: n = 30 (19f), 82.3 ± 4.3			
Grigorova-Petrova et al. (2015)/Bulgaria	pre/post intervention study	n = 10 (8f), 80.6 ± 7.3	No experience with programs with activity promoting video games; ability to follow instructions; ability to walk independently with or without assistive device more than 10 m	Severe sensory deficits; visual or hearing impairment that does not allow possibility of interaction; ataxia or other cerebellar symptoms; severe deformities or locomotion problems; uncontrolled hypertension; decompensated diabetes; high functional class heart failure	Not reported, only “institutionalized older individuals”
Huang et al. (2021)/Taiwan	cross-sectional explanatory study	n = 71 (23f), 79.1 ± 7.7	65 years old or older; able to operate two 3D VR joysticks; no experience of 3D VR	Mental disorders; significant visual or hearing impairment; dementia	Large-scale (>200-beds) long-term care facilities
Hutchinson et al. (2018)/Australia	Feasibility study	n = 4	Not reported	Not reported	Residential aged care facility
Janssen et al. (2013)/Netherlands	CT	Median (interquartile range)	Able to play the game without physical support; medically fit (determined by the activity worker of the nursing home)	Bad vision; bedridden or wheelchair-bound; cognitive impairment (score of 22 or less on the MMSE)	Nursing home
		IG1: n = 8 (4f), 84.5 (5.0)			
		IG2: n = 8 (6f), 81.5 (12.8)			
		CG: n = 13 (10f), 80.0 (8.5)			
Kamińska et al. (2018)/Poland	pre/post intervention study	n = 23 (19f), 75.7 ± 8.1	Sufficient intellectual capacity; over 60 years of age; completion of the 6MWT; scored 19–24 on the DGI; scored over 10 s on the TST; scored 27 points or less on the BDI	lack of sufficient health to participate in the study; concomitant diseases that would be a threat to the patient’s health during physical exercise	Residents of social welfare institution
Keogh et al. (2012)/New Zealand	pre/post intervention study (mixed-methods)	n = 11 (6f), 81 ± 6	>70 years of age; sufficient dexterity to manipulate the controller; cognitive ability to understand instructions, verbally communicate and complete questionnaires	Not reported	Nursing home
Keogh et al. (2014)/Australia	CT (mixed-methods, pilot study)	IG: n = 19 (17f), 81 ± 7	Ability to walk at least 10 m unaided or with a walking aid; sufficient cognitive ability to understand the instructions; ability to answer questionnaires; cognitive ability was determined based on the most recent cognitive assessment of the residents via tools such as the MSSE	Not reported	Residential aged-care centers
		CG: n = 15 (13f), 85 ± 7			
Loggia et al. (2021)/France	RCT (cross-over)	n = 12 (2f), 75.3 ± 8.5	Age >65; ability to cycle	Unstable heart disease; normal to serve limitation of activity according to the New York Heart Association; severe cognitive or physical impairment with inability to follow simple instructions; severe visual disturbance	Nursing home
Marinus et al. (2021)/Belgium	RCT (cross-over)	n = 10 (6f), 80.5 ± 5.7	Age >75; MoCa: 18–25; slowness and/or muscle weakness according to the frailty phenotype of Fried; living at least 1 month in the residential care center; able to understand simple instructions; ability to actively exercise on an exercise bike	Neurological or orthopedic disease from which progression due to the disease process could be expected on short term; blind or uncorrected visual impairment	Residential care setting
Monteiro-Junior et al. (2017)/Brazil	RCT (pilot study)	IG: n = 9 (6f), 85 ± 8, (range: 68–96)	Medical consent; independent ambulation; comprehension of simple commands and capacity of communication	Severe cardiorespiratory impairment; acute musculoskeletal injury; delirium; moderate to severe degree of diagnosed dementia	Not reported, only „institutionalized older adults”
		CG: n = 9 (6f), 86 ± 5 (range: 76–93)			
Pichierri et al. (2012)/Switzerland	RCT	IG: n = 11 (8f), 86.9 ± 5.1	Older than 65; score of at least 22 in the MMSE; able to walk at least 8 m with or without a walking aid; no rapidly progressive or terminal illness; acute illness or unstable chronic illness	Severe impairment of vision	Hostels for the aged
		CG: n = 11 (10f), 85.6 ± 4.2			
Ramnath et al. (2021)/South Africa	Cluster-randomized controlled trial	IG: n = 23, 70.8 ± 4.52	65 or older; subjective or objective memory complaints; subjective memory complaints; objective memory complaints; intact daily function; normal cognitive function; non-dement	Specific health impairments like Diabetes or Parkinson; orthopedic injuries	Retirement homes
		CG: n = 22, 74.14 ± 5.8			
Rica et al. (2020)/Brazil	RCT	n = 50 (50f), over 60 years	Over 60 years	Recent hospitalization; symptomatic cardiorespiratory disease; hypertension or uncontrolled metabolic syndrome; severe renal or hepatic disease; cognitive impairment; progressive and debilitating disease; marked obesity resulting in inability to exercise; recent bone fractures; positive reaction to ischemia during exercise test	Not reported, only “institutionalized older adults”
Rogan et al. (2016)/Switzerland	RCT	IG: n = 16 (10f), 90.4 ± 6.9 (range: 77–100)	Age over 65 years; able to stand with or without walking aids; being classified as RAI performance level >0; having a score ≥18 in the MMSE; ≤6 points on the SPPB	Visual disturbances; lower or upper leg prosthesis; acute joint disease; acute thrombosis; acute fractures; acute infections; acute tissue damage; acute surgical scars; alcohol abuse	Long-term care facility
		CG: n = 14 (10f), 87.2 ± 5.0 (range: 79–97)			
Sun et al. (2018)/China	RCT	IG: n = 30 (18f), 78.03 ± 4.32	Age 65–85 years; at least one fall in the past year; no regular fitness training; ability to walk (independently or with a walker); mental ability to accurately understand movements and instructions during training	Severe visual, hearing or speech impairment; severe bilateral lower limb bone and joint disease or trauma; neurological disease; vestibular or cerebellar dysfunction; severe cardiovascular disease; lower limb disability; observation period after surgery for malignancy, etc.; cognitive impairment; acute or chronic illness	Nursing home
		CG: n = 30 (19f), 77.73 ± 3.98			
Taylor et al. (2018)/New Zealand	Cluster-randomized controlled trial	Median (interquartile range)	Aged 65 and over; ability to move around independently with or without a walking aid; able to understand instructions for carrying out the study	Acute illness; impaired vision so that they could not see a large television screen	Low-level dependency long-term care facilities
		IG: n = 26 (20f), 86.75 (11.2)			
		CG: n = 32 (23f), 85.8 (8.1)			
Ulbrecht et al. (2012)/Germany	pre/post intervention study (pilot study)	n = 79 (53f), 79.3 ± 10.4	No acute worsening of symptoms during screening	Bedridden; blind; paralysis of arms or hands (unilateral and bilateral)	Old people home
Wu et al. (2019)/Taiwan	CT (pilot study)	IG: n = 8 (4f), 82.8 ± 9.1	Age >65 years; ability to follow simple instructions; ability to walk independently for at least 20 m with or without assistance	Unstable angina/epilepsy; severe visual impairment; cognitive impairment (score of ≤23 on the MMSE); musculoskeletal conditions that limit mobility	Long-term care facility
		CG: n = 9 (1f), 80.3 ± 6.0			
Yesilyaprak et al. (2016)/Turkey	RCT	IG: n = 7 (3f), 70.1 ± 4.0	Age of at least 65 years; at least one fall in the last year; ability to walk 10 m (with assistance if needed); ability to stand independently for 90 s	Severe visual or hearing impairment; impaired cognitive status (MMSE score <21); neurological disease; previous lower limb injury or surgery	Nursing home
		CG: n = 11 (9f), 73.1 ± 4.5			
Zahedian-Nasab et al. (2021)/Iran	RCT	IG: n = 30 (8f), 69.67 ± 7.7	Ability to walk with or without aids and permission from the nursing home doctor	History of acute or chronic physical, cognitive, and mental illness; participation in other exercise similar to intervention; unchanged hearing and vision problems; balance problems due to vestibular system and cerebellum problems	Nursing home
		CG: n = 30 (8f), 72 ± 7.8			

IG: intervention group; CG: Control/comparison group; MMSE: Mini-Mental-State Examination; FAC: functional ambulation category; 6MWT: 6-Minute Walk Test; DGI: dynamic gait index; TST: tandem stance test; BDI: beck depression inventory; RAI: resident assessment instrument; NWS: nintendo wii system; VR: virtual reality; f: female; SPPB: short physical performance battery; MoCa: Montreal Cognitive Assessment; TUG: Timed Up and Go Test. unless stated otherwise, age is presented as mean ± SD.

**TABLE 3:** Intervention/control characteristics and results of primary outcomes.

Study	Intervention Description	Duration of Intervention, Frequency, and Duration of Exercise Sessions	Control/Comparison	Results
Babadi and Daneshmandi, (2021)	Exergaming (Xbox Kinect)	9 weeks, 3x per week, 60-min	CG: Usual activities of daily living	Falls/fall Risk: Sig. improvement of IG1 compared to CG in TUG (*p* = 0.014) and FAB (*p* = 0.010); IG1 vs IG2: no sig. differences
			IG2: Conventional balance training	
Brandt and Paniagua, (2011)	Exergaming (Nintendo Wii)	4 weeks, at least once per week	No CG	Acceptability/Feasibility^a^: Wii bowling was somewhat easy or very easy to learn (81.8%), very enjoyable (60%), half of the residents preferred playing Wii to other recreational activities
Chan et al. (2001)	Nursing services provided via telemedicine	One year, not reported	No CG	Falls/fall Risk: Mean proportion of falls resulting in fractures reduced from 8 to 3%; mean number of falls reduced from 9.8 to 6.8 per month; both not significant (*p* > 0.05)
Cicek et al. (2020)	Exergaming (Nintendo Wii Fit+)	8 weeks, 2x per week, 30 min	CG: Usual activities of daily living	Falls/fall Risk: Sig. improvements of IG1 compared to CG in BBS (*p* = 0.001) and TUG (*p* = 0.001); Sig. improvements of IG1 compared to IG2 in TUG (*p* = 0.007), no sig. differences in BBS (*p* = 0.917)
			IG2: Stationary cycling and treadmill walking	
de Bruin et al. (2020)	Whole-body vibration with varying frequency, from weeks five to eight additionally exergaming (dance video game with step plate, game: StepMania 4.0, projected on a wall using a video beamer) after the vibration sessions	8 weeks, 3x per week, 10 min vibration (5 min training, 5 min break), 5 min additional dance video game from week 5 onwards	1 Hz vibration and passive trampoline-program (expected to have no effects)	Acceptability/Feasibility^a^: Adherence rate IG: 76.5%
				Falls/fall Risk: No sig. improvement compared to CG in SPPB (*p* = 0.055)
Delbroek et al. (2017)	Exergaming (BioRescue)	6 weeks, 2x per week, gradually increased from 18 min in week 1–30 min in week 5	Usual activities of daily living	No between-group comparison conducted
				Falls/fall Risk: Sig. pre-post-change for IG in iTUG total duration (*p* < 0.05), iTUG: turn to sit duration (*p* < 0.05), iTUG: turn: step-time before turn (*p* < 0.05); No sig. pre-post-change for IG in Tinetti Scale, iTUG turn: duration, iTUG: sit to stand: duration
				Acceptability/Feasibility^a^: IMI: Value-usefulness 6.4 ± 0.8, Interest-enjoyment: 6.2 ± 0.4, Perceived competence: 5.5 ± 0.5
Fakhro et al. (2020)	Exergaming (Nintendo Wii fit)	8 weeks, 3x per week, 40 min	Usual activities of daily living	Falls/fall Risk: Sig. improvements for IG compared to CG in TUG (*p* = 0.000)
Fu et al. (2015)	Exergaming (Nintendo Wii Fit balance board)	6 weeks, 3x per week, 60 min	Conventional balance training	Falls/fall Risk: Sig. improvements for IG compared to CG in S-PPA z-scores (*p* = 0.004); Number of falls sig. lower in IG compared to CG (*p* < 0.001)
Grigorova-Petrova et al. (2015)	Exergaming (Xbox 360 Kinect)	4 weeks, 5x per week, 19 min 1st week, 27 min 2 nd week, 40 min 3rd week, 40 min 4th week	No CG	Falls/fall Risk: Sig. pre-post-change in BBS (*p* < 0.005) and TUG (*p* < 0.001)
Huang et al. (2021)	3D VR with head-mounted-displays (HMD) and joysticks	9 weeks, as many times as possible	No CG	Acceptability/Feasibility^a^: Average frequency of use: 12.11 ± 1.35 sessions, continuance usage intention mean score: 13.06 ± 2.12 (score range: 3–15)
Hutchinson et al. (2018)	Exergaming (Jintronix Rehabilitation System)	6 weeks, 3x per week, 20 min duration was extended according to resident capacity, with some residents completing 45 min sessions and adding additional sessions in the program	No CG	Acceptability/Feasibility^a^: Exergaming program was rated as always enjoyable by 75% of residents at the end of the first week of intervention, 100% at the end of intervention; Adherence rate of 98.5%
				Physical Activity: Total active time during exergaming sessions from the start to the end of the intervention increased markedly
Janssen et al. (2013)	Exergaming (Nintendo Wii Fit Plus) IG1 and IG2 received same intervention	12 weeks, 2x per week, 60 min (playing time of 10–15 min per participant)	Usual activities of daily living	Falls/fall Risk: No sig. differences between groups in BBS (*p* = 0.89)
				Physical Activity: Sig. improvements for IG1 (*p* = 0.014) and IG2 (*p* = 0.005) compared to CG in PA (LAPAQ); No sig. differences between IG1 and IG2 in PA
				Acceptability/Feasibility^a^: Adherence rate IG 1: 93.8%, IG2: 87.5%
Kamińska et al. (2018)	Exergaming (Xbox 360 Kinect)	30 days, 3x per week, 30 min	No CG	Falls/fall Risk: Sig. pre-post-change in DGI (*p* = 0.008)
Keogh et al. (2012)	Exergaming (Nintendo Wii Sports)	5 weeks, at any time residents wanted to play	No CG	Falls/fall Risk: No sig. pre-post-change in FSST (*p* = 0.841)
				Acceptability/Feasibility^a^: Mean Nintendo Wii playing time per week: ∼28 min; five individuals averaged between 44 and 69 min, the remaining six averaged 1–21 min per week
Keogh et al. (2014)	Exergaming (Nintendo Wii Sports)	8 weeks, at any time residents wanted to play	Usual activities of daily living	Physical Activity: Sig. improvements for IG compared to CG in PA (RAPA) (*p* = 0.009, d = 1.19)
				Falls/fall Risk: No sig. differences between groups in FSST (*p* = 0.199)
				Acceptability/Feasibility^a^: Mean Nintendo Wii playing time in IG 30 ± 24 min (range = 1–105 min) per week
Loggia et al. (2021)	Cybercycling (a cycling course was projected on a large screen in front of the participants on which they would pedal and steer on the handlebars)	two sessions with an interval of 1–7	No CG	Physical Activity: Sig. higher cycling distance on device with VR than without (*p* < 0.01)
		days between sessions		Acceptability/Feasibility^a^: Eight participants preferred VR (67%), two preferred without VR (17%), one preferred classic ergometer (8%) and one preferred none of the options (8%)
Marinus et al. (2021)	Cybercycling (MemoRide software combines an exercise bike with Google Street View, images shown on TV) (MR)	4 weeks, 1x per week, as long (maximum 30 min) as possible	1. rest condition (participants rested for 30 min, seated in the chair or in their wheelchair)	Physical Activity: No sig. differences in cycling distance between cybercycling and cycling while TV watching and between cybercycling and cycling while TV-Off
			2. Cycling while TV watching	Acceptability/Feasibility^a^: IMI interest/enjoyment: sig. higher for the TV watching (*p* = 0.01) and cybercycling intervention (*p* = 0.04) versus rest condition; IMI pressure/tension: sig. lower during cybercycling versus TV-Off (*p* = 0.03)
			3. Cycling with TV-Off	
Monteiro-Junior et al. (2017)	Exergaming (Nintendo Wii, Wii Fit Plus and EA Sports Active)	6–8 weeks, 2x per week, 30–45 min	CG performed the same exercises as IG, but without virtual reality stimulation	Falls/fall Risk: Sig. improvements for IG compared to CG in 8UG (*p* = 0.01, d = 0.85)
Pichierri et al. (2012)	Conventional strength and balance training combined with exergaming (Dance video game (modified version of StepMania) with dance pads, screen projected on a white wall)	12 weeks, 2x per week, 40 min conventional training and additionally 10–14 min exergaming	CG performed only the strength and balance exercises	Acceptability/Feasibility^a^: Adherence rate IG: 94.7%, CG: 86.9%
Ramnath et al. (2021)	Exergaming (X-Box Kinect Sports)	12 weeks, 2x per week, 60 min (30 min playing time, 30 min break)	Group-based low intensity conventional exercise (mainly strength training, some balance training)	Falls/fall Risk: Sig. improvements for IG compared to CG in TUG (*p* < 0.001, ηp^2^ = 0.35)
Rica et al. (2020)	Exergaming (X-Box Kinect)	12 weeks, 3x per week, 60 min	Played board games	Falls/fall Risk: Sig. improvements of IG compared to CG in 8UG (*p* < 0.001)
Rogan et al. (2016)	Whole-body vibration with frequency of 3–6 Hz, combined with exergaming (Dance video game (modified version of StepMania) with dance pads, screen projected on a white wall) after the vibration sessions	8 weeks, 3x per week, 10 min vibration, 9.5–13.5 min dance video game	WBV with frequency of 1 Hz and a noise level of 1 (expected to have no training effect)	Acceptability/Feasibility^a^: Adherence rate IG: 100%
				Falls/fall Risk: Sig. improvements of IG compared to CG in SPPB (*p* = 0.004, η^2^ = 0.26)
Sun et al. (2018)	Exergaming (VR rehabilitation training system Moxun)	12 weeks, 3 per week, 50 min	Conventional falls prevention training: mainly training of balance, trunk stability, lower limb muscle strength and walking ability	Falls/fall Risk: Sig. improvements for IG compared to CG in TUG and Tinetti-Scale (*p* < 0.05); Number of falls sig. lower in IG compared to CG (*p* < 0.05)
Taylor et al. (2018)	Exergaming (X-Box Kinect)	8 weeks, 2x per week, 35 min	Usual activities of daily living, including participation in any PA sessions that they would normally attend	Acceptability/Feasibility^a^: Adherence rate IG: 55%
				Physical Activity: No significant difference between IG and CG in PA levels after intervention (*p* = 0.42)
Ulbrecht et al. (2012)	Exergaming (Nintendo Wii Sports)	6 weeks in one nursing home, 11 weeks in the 2 others, once per week	No CG	Acceptability/Feasibility^a^: Acceptance rate of exergaming: 21%; Residents who accepted the exergames were significantly younger (*p* = 0.032) and showed less cognitive impairment (*p* < 0.001)
Wu et al. (2019)	Exergaming (X-Box Kinect) combined with conventional strength and balance training	12 weeks, 2x per week, 90 min, ratio between conventional training and exergaming not reported	Usual activities of daily living	Falls/fall Risk: Sig. improvements of the IG compared to CG in TUG (*p* = 0.035); No sig. differences between groups in BBS (*p* = 0.731)
Yesilyaprak et al. (2016)	Exergaming (BTS NIRVANA (Virtual-Reality-System for rehabilitation, contains exergaming-elements))	6 weeks, 3x per week, 45–55 min	Conventional balance training	Acceptability/Feasibility^a^: Adherence rate IG: 96%, CG: 87%
				Falls/fall Risk: No sig. improvements of IG compared to CG in BBS (*p* = 0.13) and TUG (*p* = 0.23)
Zahedian-Nasab et al. (2021)	Exergaming (X-Box Kinect Sports 1 and 2)	6 weeks, 2x per week, 30–60 min	Usual activities of daily living	Falls/fall Risk: Sig. improvement of IG compared to CG in TUG (*p* < 0.001) and BBS (*p* < 0.001)

a Qualitative findings are only reported in the continuous text in the results section.

IG: intervention group; CG: Control/comparison group; DGI: dynamic gait index; VR: virtual reality; SPPB: short physical performance battery; TUG: timed up and go test; BBS: berg balance scale; FSST: four square step test; 8UG: 8-Foot Up and Go test; S-PPA: Short-form Physiological Profile Assessment; IMI: Intrinsic Motivation Inventory. unless stated otherwise, values are presented as mean ± SD.

### 3.4 Results of Individual Studies and Synthesis of Results

Results of the e- and m-health intervention studies on primary outcomes can be found in Table 3 together with intervention and control characteristics. An overview of the studies examining the secondary outcomes is provided in Table 4.

**TABLE 4:** Results of secondary outcomes.

Study	Physical Function	Cognitive Function	Psychosocial Status	Neuropsychiatric Symptoms
Babadi and Daneshmandi, (2021)	Sig. improvement of IG1 compared to CG in FRT (*p* = 0.019) and all four conditions of SLS	-	-	-
	IG1 vs IG2: no sig. differences			
Cicek et al. (2020)	Sig. improvements of IG1 compared to CG in 10MWT-fast velocity (*p* = 0.003)	-	-	No sig. differences between groups in HRSD (*p* = 0.038)
	No sig. differences between groups in 10MWT-self-selected velocity (*p* = 0.179)			
de Bruin et al. (2020)	-	Sig. improvement compared to CG in TMT B (*p* = 0.002, η^2^ = 0.78)	No sig. improvement compared to CG in FES-I (*p* = 0.334)	-
		No sig. improvement compared to CG in TMT A (*p* = 0.248) and TMT B-A (*p* = 0.621)		
Delbroek et al. (2017)	-	No between-group comparison conducted	-	-
		No sig. pre-post-change for IG and CG in MoCa		
Fakhro et al. (2020)	No sig. differences between groups in COP displacements (static balance) (*p* = 0.605)	-	-	-
Grigorova-Petrova et al. (2015)	-	Sig. pre-post-change in MMSE (*p* < 0.005)	-	-
Kamińska et al. (2018)	Sig. pre-post-change in 6MWT (*p* < 0.001), grip strength left hand (*p* = 0.043) and TST (*p* < 0.001)	-	-	Sig. pre-post-change in BDI (*p* < 0.001)
	No sig. pre-post-change in grip strength right hand (*p* = 0.106)			
Keogh et al. (2012)	-	-	No sig. pre-post-change in MFES (*p* = 0.273) and health-related QOL SF-36 physical (*p* = 0.185) and mental (*p* = 0.608)	-
Keogh et al. (2014)	Sig. improvements for IG compared to CG in bicep curl (*p* = 0.038, d = 0.65)	-	Sig. improvements for IG compared to CG in WHOQOL-BREF psychological (*p* = 0.012, d = 0.74)	-
			No sig. differences between groups in WHOQOL-BREF physical (*p* = 0.096), social (*p* = 0.483) and environmental (*p* = 0.804)	
Monteiro-Junior et al. (2017)	No sig. differences between groups in chair stand test (*p* = 0.24), arm curls (*p* = 0.09) and for all gait outcomes	Sig. improvements for IG compared to CG in DSF (*p* = 0.05, d = 1.5)	No sig. differences between groups in FES-I (*p* = 0.23)	No sig. differences between groups in GDS (*p* = 0.52)
		No sig. differences between groups in MMSE (*p* = 0.30), DSB (*p* = 1.00), VFT (*p* = 0.59), TMT (*p* = 0.14), FMTrou (*p* = 0.30), FMTrec (*p* = 0.71)		
Pichierri et al. (2012)	Sig. improvements for IG compared to CG in gait velocity (*p* = 0.041, r = 0.45) and for single support time (*p* = 0.029, r = 0.48) during fast walking dual task condition	-	No sig. differences between groups in FES-I (*p* = 0.134)	-
	No sig. differences between groups in foot placement accuracy parameters			
Ramnath et al. (2021)	Sig. improvements for IG compared to CG in 6MWT (*p* = 0.017, ηp^2^ = 0.13), FRT (*p* = 0.000, ηp^2^ = 0.35)	Sig. improvements for IG compared to CG in MMSE (*p* = 0.005, ηp^2^ = 0.18) and correct responses on Stroop Task (*p* = 0.028, ηp^2^ = 0.11)	-	-
		No sig. differences in N-Back Task parameters		
Rica et al. (2020)	Sig. improvements of IG compared to CG in arm curl, sit-and-reach test, chair stand test, 800-m walk test and SLS (for all *p* < 0.001)	-	Sig. improvements of IG compared to CG in WHOQOL-BREF (*p* < 0.001)	Sig. improvements of IG compared to CG in BDI (*p* < 0.001)
Rogan et al. (2016)	Sig. improvements of IG compared to CG in IRFD right and left leg extension and right and left leg flexion manoeuvres (*p* < 0.05)	-	-	-
	No sig. differences in IMVC measures			
Sun et al. (2018)	Sig. improvements for IG compared to CG in 30sCST and static balance (for all *p* < 0.05)	-	Sig. improvements for IG compared to CG in MFES (*p* < 0.05)	-
Taylor et al. (2018)	No sig. improvement in DEMMI scores for IG (*p* = 0.06)	-	-	-
Ulbrecht et al. (2012)	-	No sig. improvements in DemTect (*p* = 0.360) and MMSE (*p* = 0.281)	-	-
Wu et al. (2019)	Sig. improvements of IG compared to CG in handgrip strength (*p* = 0.035)	-	-	-
	No sig. differences between groups in shoulder abduction (*p* = 0.445), elbow flexion (*p* = 1.000), hip flexion (*p* = 0.073), knee extension (*p* = 0.836) and ankle dorsiflexion strength (*p* = 1.000), as well as 6MWT (*p* = 1.000) and 10MWT (*p* = 0.101)			
Yesilyaprak et al. (2016)	No sig. improvements of IG compared to CG in the four conditions of the SLS and the two conditions of the TS	-	No sig. improvements of IG compared to CG in FES-I (*p* = 0.63)	-
Zahedian-Nasab et al. (2021)	-	-	Sig. improvement of IG compared to CG in FES-I (*p* < 0.001)	-

IG: intervention group; CG: Control/comparison group; MMSE: Mini-Mental-State Examination; 6MWT: 6-Minute Walk Test; DGI: dynamic gait index; TST: tandem stance test; BDI: beck depression inventory; MoCa: Montreal Cognitive Assessment; TS: tandem stance, FES-I: falls efficacy scale international; SLS: Single-leg stance; 10MWT: 10-m walk test, DEMMI: de Morton Mobility Index; DSF: digit span forward; DSB: digit span backward; VFT: verbal fluency test; TMT: trail making test; MFES: modified falls efficacy scale; 30sCST: 30-s Chair Stand Test; IRFD: isometric rate of force development; IMVC: isometric maximal voluntary contraction; FMTrou: Floor Maze Test, route; FMTrec: Floor Maze Test, recall; WHOQOL: WHO, quality of life.

#### 3.4.1 Physical Activity

Six studies reported PA related outcomes, of which only three reported data on overall PA. Of these, two studies assessed overall PA subjectively, one (Janssen et al., 2013) using the Longitudinal Aging Study Amsterdam Physical Activity Questionnaire (LAPAQ, Stel et al., 2004) and one (Keogh et al., 2014) the Rapid Assessment of Physical Activity (RAPA, Topolski et al., 2006). Both reported significant improvements in favor of the Nintendo Wii exergaming intervention groups compared to control conditions. In the study by Janssen et al. (2013), both intervention groups (Group 1 already had experience with the Nintendo Wii, Group 2 were novices) significantly increased their total PA by approximately 60 min/day compared to the control group. The greater increase in PA levels for the Nintendo Wii group compared to the control group in the study of Keogh et al. (2014) seems to be due to the transition of one individual initially categorized as “sedentary” to the RAPA category “insufficiently active” and two “insufficiently active” individuals to the “active” category. The study that examined PA objectively (Taylor et al., 2018) used body-worn sensors for 3 days each before and after the intervention. PA was defined by the authors as the percentage of time spent in an upright position (standing or walking) during the waking day. No significant differences in PA levels were observed between exergaming and control group after the intervention. Unfortunately, the authors did not report any data on PA levels beside the *p*-value.

The three remaining studies only reported data on differences in PA across exercise sessions. Hutchinson et al. (2018) reported an increase in total active time during exergaming sessions from the start to the end of the intervention. Two cross-over studies compared the cycling distance in cybercycling with normal cycling on an ergometer. While Marinus et al. (2021) found no significant differences, Loggia et al. (2021) reported that cycling distance and cycling duration were significantly higher in the cybercycling condition.

#### 3.4.2 Falls and Fall Risk

Twenty-one studies reported outcomes on falls and fall risk. Of those, three assessed the number of falls, while nineteen employed performance-oriented balance tests as a proxy measure of fall risk.

In the study where a fall prevention program was provided via videoconferencing, the mean number of falls declined from 9.8 (during the 6 months preceding the study) to 6.8 falls per month during the 12-months intervention period (Chan et al., 2001). Additionally, the mean proportion of falls resulting in fractures declined from 8 to 3%. However, the changes were not significant. Of the exergaming interventions, two studies reported a number of falls. In the study of Fu et al. (2015), the number of falls (year before intervention vs over the 12-month period after randomization) decreased in both the conventional balance group and the exergaming group. The decrease in the exergaming group was found to be significantly higher. Similar results were observed by Sun et al. (2018) who found a significant decrease in the conventional falls prevention training as well as in the exergaming group when comparing the number of falls the year before the study with the year after completion of the study. Moreover, the exergaming group showed a significant decrease compared with conventional training.

To assess fall risk, performance-oriented balance tests are commonly used. All the tests below were described to be able to identify individuals who are prone to falls or to predict the probability of falls in various populations (Shumway-Cook et al., 2000; Hall et al., 2004; An et al., 2017; Cleary and Skornyakov, 2017; Raîche et al., 2000; Jeon and Kim, 2017; Lauretani et al., 2019; Rose et al., 2002). Nine studies (Grigorova-Petrova et al., 2015; Yesilyaprak et al., 2016; Sun et al., 2018; Wu et al., 2019; Cicek et al., 2020; Fakhro et al., 2020; Babadi and Daneshmandi, 2021; Ramnath et al., 2021; Zahedian-Nasab et al., 2021) assessed fall risk using the Timed Up and Go Test (TUG, Mathias et al., 1986), two (Monteiro-Junior et al., 2017; Rica et al., 2020) a slightly modified version of the TUG, the 8-Foot Up and Go test (8UG, Rikli and Jones, 1999), and one (Delbroek et al., 2017) the instrumented TUG (iTUG, Salarian et al., 2010), which uses portable inertial sensors for a more detailed analysis. All studies reporting TUG scores found significant improvements for the exergaming intervention groups. When compared to groups that received a combination of balance and strength training (Sun et al., 2018; Ramnath et al., 2021) or bike ergometer and treadmill training (Cicek et al., 2020) the exergaming groups improved significantly. However, compared to conventional balance training without strength exercises, no differences were observed (Yesilyaprak et al., 2016; Babadi and Daneshmandi, 2021). The two studies assessing fall risk with the 8UG also reported positive effects. Monteiro-Junior et al. (2017) found a significant improvement compared to the comparison group that performed the same exercises as the exergaming group, but without the virtual reality stimulation. Rica et al. (2020) found a significant improvement compared the control group that only played board games. The iTUG results showed that total time, turn-to-sit transition, and the step-time before the turn significantly decreased for the exergaming intervention pre-to post-measurement, while neither sit-to-stand transition nor turn duration changed. No changes were revealed in the control group which received usual care (Delbroek et al., 2017).

Six studies (Janssen et al., 2013; Grigorova-Petrova et al., 2015; Yesilyaprak et al., 2016; Wu et al., 2019; Cicek et al., 2020; Zahedian-Nasab et al., 2021) assessed fall risk with the Berg Balance Scale (BBS, Berg, 1989). Here, the results of the studies were heterogeneous. Neither Janssen et al. (2013) nor Wu et al. (2019) found a significant improvement compared to the usual care control group, whereas Cicek et al. (2020) and Zahedian-Nasab et al. (2021) did. In comparison to conventional exercise, no differences were observed (Yesilyaprak et al., 2016; Cicek et al., 2020). Additionally, three studies reported significant improvements when comparing pre-to post-measurements (Grigorova-Petrova et al., 2015; Yesilyaprak et al., 2016; Zahedian-Nasab et al., 2021).

Other performance-oriented balance tests evaluating the risk of falls were the Short Physical Performance Battery (SPPB, Guralnik et al., 1994) used in two studies (Rogan et al., 2016; de Bruin et al., 2020), the Tinetti Scale (TS, Tinetti, 1986, sometimes referred to as Performance Oriented Mobility Assessment) also used by two studies (Delbroek et al., 2017; Sun et al., 2018), the Fullerton Advanced Balance scale (FAB, Rose et al., 2006) in Babadi and Daneshmandi (2021), the Four Square Step Test (FSST, Dite and Temple, 2002) in Keogh et al. (2012) and Keogh et al. (2014), the Dynamic Gait Index (DGI, Shumway-Cook and Woollacott, 2007) in Kamińska et al. (2018) and the Short-form Physiological Profile Assessment (S-PPA, Lord et al., 2003) in Fu et al. (2015).

Five studies (Fu et al., 2015; Kamińska et al., 2018; Sun et al., 2018; de Bruin et al., 2020; Babadi and Daneshmandi, 2021) reported significant improvements for the exergaming group post intervention, while two (Keogh et al., 2012; Delbroek et al., 2017) did not. When compared to usual care (Keogh et al., 2014; Babadi and Daneshmandi, 2021) or sham interventions, which were expected to have no effects (Rogan et al., 2016; de Bruin et al., 2020), heterogenous effects were reported. Two studies (Rogan et al., 2016; Babadi and Daneshmandi, 2021) found a significant improvement compared to control, while the others (Keogh et al., 2014; de Bruin et al., 2020) did not. However, in the study of de Bruin et al. (2020), the small sample size and the *p*-value (0.055) suggest that more data would be needed for definite interpretation. In the study by Keogh et al. (2014), residents chose the frequency and duration of exergaming, resulting in only 30 min of exergaming per week on average. Compared with conventional training, two studies found significant improvements (Fu et al., 2015; Sun et al., 2018) while Babadi and Daneshmandi, (2021) did not.

#### 3.4.3 Acceptability and Feasibility

Adherence was reported by seven studies. Five of them (Pichierri et al., 2012; Janssen et al., 2013; Rogan et al., 2016; Yesilyaprak et al., 2016; Hutchinson et al., 2018) showed adherence rates to exergaming sessions of 91% or more. This contrasts with values of 55% (Taylor et al., 2018) and 76.5% (de Bruin et al., 2020). Compared to groups with conventional balance training (Yesilyaprak et al., 2016) or sham control group (Pichierri et al., 2012), adherence rates were lower in the non-exergaming groups (both studies: 87%). Two studies did not provide fixed exergaming sessions to the residents, instead they were free to play if and when they wanted. This resulted in a similar amount of mean playing time, i.e., 28 min per week over 5 weeks (Keogh et al., 2012) and 30 min per week over 8 weeks (Keogh et al., 2014), respectively. In both studies, the mean duration was consistent across the course of the intervention. However, considerable interindividual differences were observed, with a range of 1–69 min (Keogh et al., 2012) and 1–105 min (Keogh et al., 2014) per week, respectively.

Ulbrecht et al. (2012) studied the acceptance rate of residents living in three different NH in Germany. The therapists introduced exergames to the residents and then provided them with the games once a week for 3 weeks to find out how many participants would accept them. Acceptance was defined by the authors as the interest in continuing to play exergames after the 3 weeks. They reported an overall acceptance rate of 21% (27 of 79 residents). In one NH in which all residents were screened, an acceptance rate of 20% was observed. Residents who accepted the exergames were significantly younger and showed less cognitive impairment. The percentage of individuals with Mini-Mental-State Examination (MMSE) scores indicating dementia was 59.6% among those who refused the games and 37% among those who accepted the games. Impairment of arm or hand mobility was noted in eight (30%) of the 27 participants who accepted the exergames and in 16 (31%) of the 52 participants who rejected them. Brandt and Panigua, (2011) found that half of the residents preferred playing Nintendo Wii to other recreational activities and that negative aspects of playing Nintendo Wii were fear of falling out of the chair or, for some residents, lack of interest.

Enjoyment, as a measure of acceptability of exergaming, was reported by six studies. Delbroek et al. (2017) assessed enjoyment with the interest/enjoyment domain of the Intrinsic Motivation Inventory (IMI, Ryan, 1982). The mean score was 6.2 ± 0.4, with seven being the highest possible score. In the study by Hutchinson et al. (2018) the exergaming program was rated as “always enjoyable” by 75% of residents at the end of the first week of intervention, and this percentage increased to 100% at the end of the 6-week program. Brandt and Paniagua, (2011) reported that Wii exergaming was rated as very enjoyable by 60% of residents. Three studies assessed perceived enjoyment qualitatively. Keogh et al. (2012) stated that residents enjoyed not only the fact that their activities elicited laughter from other participants as well as observers, but also the new and strengthened friendships that were formed as a result. For some male members of the group, competition among themselves was another fun element. Both Keogh et al. (2014) and Taylor et al. (2018) reported that playing exergames was enjoyable for residents.

Furthermore, it was reported that 82% of residents found Nintendo Wii bowling somewhat easy or very easy to learn (Brandt and Paniagua, 2011). The value and usefulness domain of the Intrinsic Motivation Inventory reached a score of 6.4 ± 0.8 (Delbroek et al., 2017). In addition, the NH staff felt that exergaming is an activity that can be relatively easily integrated into the NH environment, given that there is a room with a TV and sufficient space, and that there is someone who can operate the system (Keogh et al., 2012). Hutchinson et al. (2018) found that residents were proficient in exergaming activities by the second week of intervention, while Keogh et al. (2012) reported that residents were confident in operating the Nintendo Wii at the conclusion of the 5-week intervention.

Two studies mentioned the initial concern of most residents regarding their lacking experience in dealing with technology and the fear to potentially reveal their lack of knowledge in front of others. This disappeared after residents gained confidence in using the systems and took pride in their ability to use modern technology (Keogh et al., 2012; Keogh et al., 2014). Delbroek et al. (2017) reported a score of 5.5 ± 0.5 in the Intrinsic Motivation Inventory domain perceived competence. It was noted that intervention facilities purchased a Nintendo Wii after the intervention because most residents wanted to continue exergaming (Keogh et al., 2012), and home management was impressed with the psychosocial changes in their residents (Keogh et al., 2014).

Acceptability of the 3D VR-based horticultural therapy program (Huang et al., 2021) was determined using the variables frequency of use during the nine-week free trial period and continuance usage intention. Residents did not report significant cybersickness symptoms due to the head-mounted display. One possible reason for this could be that most of them practiced VR in a seated position. The average frequency of use was 12.11 ± 1.35 sessions and 78% of residents completed the program successfully. Continuance usage intention was measured by residents’ future willingness to participate in a 3D VR program, practice the old program, or attend a new program. The total score reached a mean of 13.06 ± 2.12 (Possible score range: 3–15), with higher scores indicating a higher level of continuance usage intention.

In the cybercycling interventions it was reported that eight residents (67%) preferred cybercycling, while one (8%) was neutral (Loggia et al., 2021). Marinus et al. (2021) found that the score of the interest/enjoyment subscale of the Intrinsic Motivation Inventory was significantly higher for the TV watching and the cybercycling condition compared to the rest condition, in which participants rested for 30 min in a chair. In addition, the score of the pressure/tension subscale was significantly lower during cybercycling compared to the TV off condition.

#### 3.4.4 Secondary Outcomes

Since none of the other e-health interventions examined any secondary outcome, only results for the effects of exergaming are presented in the following.

##### 3.4.4.1 Psychosocial Status

Seven studies reported falls efficacy. Five (Pichierri et al., 2012; Yesilyaprak et al., 2016; Monteiro-Junior et al., 2017; de Bruin et al., 2020; Zahedian-Nasab et al., 2021) used the Falls Efficacy Scale-International (FES-I, Yardley et al., 2005), while two (Keogh et al., 2012; Sun et al., 2018) employed the Modified Falls Efficacy Scale (MFES, Hill et al., 1996). No improvement compared to control groups was found in four studies (Pichierri et al., 2012; Yesilyaprak et al., 2016; Monteiro-Junior et al., 2017; de Bruin et al., 2020) while two studies observed a significant effect (Sun et al., 2018; Zahedian-Nasab et al., 2021). A significant improvement when comparing pre-to post-measurements was reported in three studies (Pichierri et al., 2012; Sun et al., 2018; Zahedian-Nasab et al., 2021), whereas two found no differences (Keogh et al., 2012; Yesilyaprak et al., 2016).

Four studies investigated the effect of exergaming on quality of life. Three (Keogh et al., 2014; Cicek et al., 2020; Rica et al., 2020) employed the short version of the World Health Organization Quality of Life Questionnaire (WHOQOL-BREF, WHO, 1996) and one (Keogh et al., 2012) the Short Form Health Survey questionnaire (SF-36, Ware and Sherbourne, 1992). While Cicek et al. (2020) found no significant changes in the four domains of the WHOQOL-BREF, Keogh et al. (2014) observed significantly greater improvements in psychological quality of life for the exergaming group than the control group and Rica et al. (2020) found significant improvements in all four domains compared to the control group. Keogh et al. (2012) reported a 13.3% increase in the physical health domain of the SF-36; however, this was not statistically significant.

##### 3.4.4.2 Neuropsychiatric Symptoms

Depressive symptoms were assessed using the Beck Depression Inventory (BDI; Beck et al., 1996) in Kamińska et al. (2018) and Rica et al. (2020), the Hamilton Rating Scale for Depression (HRSD, Hamilton, 1986) in Cicek et al. (2020) and the Geriatric Depression Scale (GDS, Yesavage et al., 1982) in Monteiro-Junior et al. (2017). A significant reduction in depression scores when comparing pre-to post-measurements was observed in three studies (Kamińska et al., 2018; Cicek et al., 2020; Rica et al., 2020), while one study did not report the *p*-values of within-group differences (Monteiro-Junior et al., 2017). Compared to control conditions, only one study found a significant reduction in depression scores (Rica et al., 2020), whereas Cicek et al. (2020) and Monteiro-Junior et al. (2017) did not.

##### 3.4.4.3 Cognitive Function

General cognitive function was evaluated in five studies, with four (Ulbrecht et al., 2012; Grigorova-Petrova et al., 2015; Monteiro-Junior et al., 2017; Ramnath et al., 2021) using the MMSE (Folstein et al., 1975) and one (Delbroek et al., 2017) the Montreal Cognitive Assessment (MOCA, Nasreddine et al., 2005). Ulbrecht et al. (2012) employed the DemTect (Kalbe et al., 2004) in addition to the MMSE. Significant improvement compared to the comparison group which received conventional group-based exercise was reported in one study (Ramnath et al., 2021), and Grigorova-Petrova et al. (2015) found a significant improvement in MMSE scores in the post-measurement. No significant differences were observed in the other studies.

Other cognitive tests utilized were the Trail Making Tests A (Monteiro-Junior et al., 2017; de Bruin et al., 2020) and B (de Bruin et al., 2020), the Floor Maze Test, the Digit Span Forward, Digit Span Backward, Verbal Fluency Test (Monteiro-Junior et al., 2017), as well as the N-Back Task and the Modified Stroop task (Ramnath et al., 2021). Of these, performance in the Trail-making-test B (de Bruin et al., 2020), the Digit Span Forward (Monteiro-Junior et al., 2017) and the total number of correct responses on the Stroop task (Ramnath et al., 2021) improved significantly in the exergaming compared to control groups.

##### 3.4.4.4 Physical Function

Seven studies assessed strength parameters. In Kamińska et al. (2018), grip strength of the left hand was significantly higher in the post-measurement, while there were no significant differences observed for the right hand. Wu et al. (2019) found a significant increase in hand grip strength compared to control. Biceps strength, assessed with the number of biceps curl repetitions, increased significantly compared to control in two studies (Keogh et al., 2014; Rica et al., 2020), while one study (Monteiro-Junior et al., 2017) did not observe a significant difference. Biceps strength assessed with a dynamometer also did not change significantly compared to control (Wu et al., 2019). Three studies (Monteiro-Junior et al., 2017; Sun et al., 2018; Rica et al., 2020) used chair stand tests to assess lower limb strength. Of these, two (Sun et al., 2018; Rica et al., 2020) found significant improvements compared to control, whereas one (Monteiro-Junior et al., 2017) did not. Other strength tests included shoulder abduction, hip flexion, ankle dorsiflexion and knee extension, all of which showed no significant differences (Wu et al., 2019). Additionally, Rogan et al. (2016) found significant improvements on isometric rate of force development (IRFD) of knee extension and knee flexion compared to control.

Four studies assessed static balance with single-leg stance tests. Significantly increased balance in the single-leg stance for the exergaming group compared to control not receiving an exercise intervention was observed in two studies (Rica et al., 2020; Babadi and Daneshmandi, 2021), while one did not find significant differences compared to usual care (Cicek et al., 2020). Compared to conventional balance training, no significant differences were found (Yesilyaprak et al., 2016; Cicek et al., 2020; Babadi and Daneshmandi, 2021).

Other measures of static balance were assessed in four studies. Fakhro et al. (2020) conducted a computer analysis of center of pressure displacements and found no significant differences between the exergaming and usual care control group. Sun et al. (2018) used the PC708 balance function system to assess static balance and found a significant improvement regarding the deviation of the center of pressure on the *X* and *Y* axes compared to conventional exercise. Kamińska et al. (2018) found a significant improvement after the intervention in the tandem stance test, as did Yesilyaprak et al. (2016) in the eyes closed condition. Compared to conventional exercise, no significant differences were found (Yesilyaprak et al., 2016).

Mobility scores in the de Morton Mobility Index (de Morton et al., 2008) did not improve significantly in the exergames group after intervention. Additionally, the authors reported that age had a significant effect on DEMMI scores, such that higher age was associated with lower DEMMI scores in intervention and control group. In contrast, neither gender nor cognition was a significant predictor of DEMMI scores (Taylor et al., 2018). Two studies assessed mobility with functional reach tests (Babadi and Daneshmandi, 2021; Ramnath et al., 2021) and one (Rica et al., 2020) with a sit-and-reach test. Rica et al. (2020), as well as Babadi and Daneshmandi, (2021), found significant improvements for the exergaming group compared to non-exercise control. Compared to conventional multimodal exercise, significantly increased mobility was reported (Ramnath et al., 2021), while compared to conventional balance training no significant improvements were found (Babadi and Daneshmandi, 2021).

Self-selected walking speed assessed by 10-m walk tests did not show any significant differences, neither compared to pre-intervention (Cicek et al., 2020), nor compared to usual care or conventional balance training (Wu et al., 2019; Cicek et al., 2020). However, in the fast velocity condition of the 10-m walk test, the exergaming group showed significant improvements compared to pre-intervention and usual care (Cicek et al., 2020). Monteiro-Junior et al. (2017) found in their gait analysis no significant between-group differences for step length, step variability, and mean speed.

Regarding foot placement performance, no significant differences were observed between groups, whereas significant between-group differences were found in spatio-temporal gait parameters. In the condition in which subjects had to walk as fast as possible while completing a cognitive task, the exergaming group showed a significant increase in walking speed and a decrease in single support time compared to the control (Pichierri et al., 2012).

Aerobic capacity was evaluated in four studies, three used a 6-min walking test (Kamińska et al., 2018; Wu et al., 2019; Ramnath et al., 2021), and one an 800-m walk test (Rica et al., 2020). Studies found significant improvements compared to conventional exercise (Ramnath et al., 2021) and non-exercise control group (Rica et al., 2020) as well as compared to pre-intervention (Kamińska et al., 2018; Rica et al., 2020; Ramnath et al., 2021). No significant differences were reported in Wu et al. (2019).

## 4 Discussion

This systematic review aimed at providing an overview of the effectiveness as well as the acceptability and feasibility of e- and m-health interventions in promoting PA and preventing falls in NH. Additionally, the effectiveness regarding physical and cognitive function, neuropsychiatric symptoms and psychosocial status was investigated. Remarkably, 24 of the 28 included studies investigated digital exergaming as an intervention or part of the intervention, while none incorporated a m-health intervention. Based on the included studies, the duration of the intervention does not seem to influence effectiveness, while the results indicate that interventions with three or more sessions per week tend to be more effective. Exergaming was shown to be effective in reducing the number of falls and fall risk in NH residents in most studies. Conversely, due to the limited number of studies and heterogenous results, the impact of exergaming on PA levels of NH residents seems to be unclear. It was described as feasible and well accepted by NH residents. Regarding secondary outcomes, exergaming demonstrated significant improvements, although the results were not consistent for all outcomes. The two studies on cybercycling showed contrasting results in terms of an increased cycling distance compared to conventional stationary cycling. One study with a VR-based horticultural therapy program found that the intervention was highly accepted by NH residents. The provision of a falls prevention program via videoconferencing did not lead to a significant reduction in the number of falls. In the following, results will be discussed separately for all primary and secondary outcomes.

### 4.1 Physical Activity

As only three studies, all of which were exergaming interventions, examined the impact on overall PA, it is difficult to draw any conclusions about effectiveness. Furthermore, PA was objectively measured in only one of these studies (Taylor et al., 2018). In the two remaining studies in which PA was measured subjectively (Janssen et al., 2013; Keogh et al., 2014), significant improvements were reported compared to the usual care control groups. It seems likely that the self-reported increased PA was mainly due to the training sessions during the intervention, as the questionnaires were completed shortly after the end of the intervention. Based on this, follow-up measurements examining whether NH residents’ PA levels remain elevated weeks after an exergaming intervention are needed. In contrast, in the study with objective measurement, no significant differences in PA levels after intervention were observed. However, the definition of PA as being in an upright position and the restriction of the measurement between 10a.m. and 8p.m. must be viewed critically. A review of Sween et al. (2014) reported a strong correlation between exergaming and increased energy expenditure, with up to 300% above resting levels and that most exergames have been found to elicit moderate-intensity PA, meeting the American College of Sports Medicine’s guidelines for health and fitness (Haskell et al., 2007). However, only five studies in the review by Sween et al. (2014) measured the effect of exergaming in adults. Taylor et al. (2012) reported that the nine different exergames examined in their study provided light-intensity exercise for community-dwelling older adults and no differences were observed in energy expenditure between Nintendo Wii and Xbox 360 Kinect games. In addition, no significant difference in energy expenditure was found between exergaming while standing or sitting, indicating that individuals who cannot stand may derive equivalent benefit from exergaming in a sitting position.

It remains unclear whether cybercycling causes NH residents to exercise longer and more intensively, as the two studies yield different results. In a study with residents of independent living facilities, a cybercycling intervention was found to result in greater cognitive benefits compared to a similar dose of conventional stationary cycling (Anderson-Hanley et al., 2012). However, this cybercycling intervention provided the option to compete with other participants and is therefore difficult to compare with the two studies in present review. Thus, competition should be examined as a separate factor in future studies involving cybercycling.

### 4.2 Falls and Fall Risk

Since only two exergaming intervention studies (Fu et al., 2015; Sun et al., 2018) reported effects on the number of falls, evidence is limited. However, both demonstrate robust study designs (RCTs), with strong and moderate overall study quality. Significant reductions in falls were observed in both RCTs, even when compared to conventional fall prevention training. Similarly, Stanmore et al. (2019) reported that exergaming resulted in a significant change in fall rates among older adults living in assisted living facilities. One explanation for the effectiveness of exergaming might be the visual and auditory real-time feedback that digital exergames provide, delivering stimuli which support error-free learning (Fu et al., 2015). Further research is needed to confirm these findings, but preliminary results are promising.

In most of the performance-oriented balance tests used to assess fall risk, significant improvements in exergaming groups were reported across studies. In their systematic review, Alhagbani and Williams, (2021) also measured fall risk with performance-oriented balance tests and reported a reduced risk of falls in community-dwelling older adults after home-based exergaming interventions. It should be mentioned that a single performance-oriented balance test must be interpreted with caution regarding its predictive accuracy for fall risk. Recent systematic reviews conclude that fall risk assessment tools currently used in the elderly do not have sufficiently high predictive validity to discriminate between high and low fall risk (Lima et al., 2018; Park, 2018). Therefore, it is recommended to use two or more assessment instruments. Using multiple assessment instruments that have different characteristics can increase the overall prediction accuracy (Park, 2018). Of the seven studies reporting two different measures of fall risk, six reported significant improvements for the exergaming group in both tests. Moreover, significant improvements compared to conventional exercise were reported in about half of the fall risk assessments (Cicek et al., 2020 (TUG, not in the BBS); Fu et al., 2015 (S-PPA); Monteiro-Junior et al., 2017 (8UG); Ramnath et al., 2021 (TUG); Sun et al., 2018 (TUG, TS)). However, differences were observed between the various tests. Specifically, a higher frequency of significant differences was reported for the TUG test compared to the BBS. Similarly, Suleiman-Martos et al. (2021) found in their systematic review and meta-analysis that exergaming has significant beneficial effects on the TUG compared to conventional training in community-dwelling older adults, while the effects produced on the BBS and the 8UG test did not differ significantly from those obtained with conventional exercise. Other reasons for the differences between the studies could be the content of the conventional training and the duration and frequency of the interventions, as large discrepancies existed in these aspects. The results indicate that exergaming is superior to conventional training in terms of fall risk reduction if it does not or only to a small extent contain balance training. Regarding training frequency, interventions consisting of three training sessions per week seem to be more effective than interventions with a lower training frequency which might not be able to achieve sufficient stimulus. Overall, none of the included studies used a sensor-based fall risk assessment, which has been shown to be feasible and able to successfully distinguish between groups of faller status (Bezold, Krell-Roesch et al., 2021).

It is particularly interesting that significant improvements were observed in the exergaming intervention group compared to a comparison group, although both groups performed the same physical exercises (Monteiro-Junior et al., 2017). Game elements such as competition and challenge as well as the immediate feedback, which are described by older adults as positive aspects of exergames (Nawaz et al., 2016), might be the reason for the observed differences.

Interventions in NH where residents are free to choose the frequency and duration of exergaming might not be suitable for improving performance-oriented balance (Keogh et al., 2012; Keogh et al., 2014). It seems that the induced amount of training was too small to produce effects. However, it cannot be ruled out that the FSST used only in these two studies had an impact on the results.

### 4.3 Acceptability and Feasibility

Overall, exergaming was described to be feasible for NH and high acceptability was reported among residents. It was also reported that residents enjoyed playing, mainly because of the social interaction and competition. Although it was stated that many residents were initially concerned regarding dealing with the new technology, after some time they learned to deal with the exergaming devices and gathered confidence (Keogh et al., 2012; Keogh et al., 2014).

The proportion of NH residents for whom exergaming is feasible is difficult to quantify. Ulbrecht et al. (2012) estimated that exergaming may be suitable for only one in five residents. The results of the study from Germany showed that residents who accepted the exergames were significantly less cognitively impaired and were less likely to be hearing impaired. The proportion of subjects with MMSE scores indicating dementia was 59.6% among those who declined exergaming and 37% among those who accepted it. A study from Sweden with data from 188 randomly selected NH found that 41.4% of residents show moderate or severe cognitive impairment (Björk et al., 2016). This percentage varies around the world, depending on location, country, and region (Seitz et al., 2010). However, these findings suggest that in western countries, exergaming might not be feasible for more than one-third of residents in terms of cognitive impairment only. The reason for this is that in people without or with only mild cognitive impairments, instructions and explanations on exercise and its benefits are usually used to promote PA (Spittaels et al., 2007). In the presence of advanced cognitive impairment, this approach is not feasible as individuals are unable to process and remember verbal instructions (Hill et al., 2010). However, there are innovative technology solutions specifically designed for this target group, which address the individual in a more implicit way. Braun et al. (2015) developed “interactive surfaces”, a combination of virtual reality and exergaming. Here, various graphic shapes are projected onto the floor via a projector. The surfaces are activated by the movements of a person in the projection field captured by an infrared camera. The difficulty is that developing such software specifically tailored to the requirements of the target group is expensive and complex (Pirovano et al., 2016). In addition, the acquisition of the required systems is cost-intensive for NH.

The virtual reality horticultural therapy was found to be well accepted by NH residents. However, the mean frequency of use during the free trial period was only ∼1.3 sessions per week. Moreover, the high initial cost of head-mounted displays, depending on the model, appears to be a barrier to their use in NH. Nevertheless, prices in this segment have declined significantly in recent years (Statista Research Department, 2020), which is why usage could become much more affordable in the future. Previous research has so far shown encouraging results in the use of virtual reality head-mounted displays in the rehabilitation of patients and might be a promising technology for the future (Tieri et al., 2018).

Overall, it is remarkable that we did not find a single m-health intervention targeting NH residents. Aslam et al. (2020) concluded in their systematic review that m-health interventions may be useful, acceptable, and beneficial in maintaining and improving physical activity in older adults, although little is known about the long-term effectiveness. While many NH residents, particularly those with advanced cognitive impairments, are likely to be unable to use m-health applications on their own, such an intervention aimed at helping nursing home staff to promote PA may be a viable solution (Barisch-Fritz et al., 2022b). Compared with the exergames used in the included studies, a customizable m-health application could also be more feasible to address the respective physical and cognitive abilities of NH residents.

### 4.4 Secondary Outcomes

Of the studies included, only those containing digital exergaming examined the effects on any secondary outcome. Although the results are inconsistent, the findings indicate that exergaming has the potential to positively impact cognitive functioning in NH residents. A review of the effects of exergaming on cognitive function in people with mild cognitive impairment and dementia reported more explicit results (Zhao et al., 2020). The authors concluded that there is consistent evidence with a low risk of bias showing significant positive effects of exergaming on cognitive function. The included studies had a similar mean age of 80 years, a similar mean intervention duration of 8 weeks, and comparable cognitive status. However, half of the interventions were conducted in community-dwelling adults, and median exercise frequency was with three times per week higher, which might explain the differences in results.

Based on the results of the included studies, exergaming interventions appear to significantly improve depressive symptoms among NH residents. This finding is consistent with high enjoyment reported for the interventions, as well as with a recent meta-analysis of RCTs that found a large effect of exergaming on depressive outcomes in older adults (Yen and Chiu, 2021). It is assumed that exergames have a positive impact on depressive symptoms by increasing social interaction with other residents, staff, and therapists, by creating a sense of accomplishment, and by enhancing mood (Chao et al., 2015).

Mixed results were reported regarding the effectiveness of exergaming in reducing fear of falling in NH residents. Three out of seven studies found significant improvements in fall efficacy. One reason for this might be that three of the studies reporting non-significant results had small sample sizes (maximum 9 subjects per group) and might have lacked statistical power. The non-significant results of Keogh et al. (2012) may be explained by the fact that it was one of the two included studies without fixed exergaming sessions, resulting in an average playing time of only 28 min per week. These findings are similar to those of a systematic review of the efficacy of exergames on fear of falling in community-dwelling older adults (Ge et al., 2021). It was noted that 15 of the 23 studies reported statistically significant changes in fear of falling. However, the authors concluded that the effectiveness of exergames may depend, to some extent, on the instrument used to measure fear of falling. Fear of falling measured as balance confidence with the Activities-specific Balance Scale proved to be effective in all interventions, whereas less than half of the interventions in which the fear of falling was measured as fall-related self-efficacy was found to be effective.

Regarding the quality of life, the effects of exergaming are also heterogeneous. Two studies reported no significant improvements, one only in the psychological domain and one in all domains. These differences might be explained by the fact that the study with significant effects in all domains (Rica et al., 2020) lasted with 12 weeks considerably longer than the other studies (5–8 weeks). Moreover, the intervention consisted of 180 min per week of exergaming, compared to 30–60 min per week in the other studies. Cacciata et al. (2019) also reported mixed results. Their systematic review investigated the effectiveness of exergaming on health-related quality of life in older adults aged 65 years and older. Of the nine articles included, three found significant improvements. The authors attributed the inconsistent findings partly to heterogeneity in exergaming platforms, data collection instruments, as well as duration and frequency of interventions, which is also the case for the studies included in the present review.

Exergaming was found to be effective in improving the physical function in NH residents in most studies and parameters. Especially for handgrip strength, mobility, and aerobic capacity, significant effects were reported. However, compared with conventional training, significant improvements were rarely observed. A systematic review and meta-analysis on the effects of exergaming on physical function in community-dwelling older adults found significant beneficial effects on gait speed (Suleiman-Martos et al., 2021). The effects produced on other functional parameters such as aerobic capacity (6-min walk test), lower limb strength (30-s chair stand and knee extension strength) and grip strength did not differ significantly from those obtained with conventional exercise training. Similarly, Skjæret et al. (2016) found that most studies showed comparable changes in physical function for exergaming compared with conventional training in older adults aged 65 years and above. The authors concluded that exergaming might be an effective alternative to conventional exercise for older adults.

### 4.5 Commercial Exergames vs Specifically Designed Exergames for Rehabilitation

We did not observe differences in the effectiveness of exergaming devices when comparing commercial exergaming platforms such as Nintendo Wii and X-Box Kinect to exergaming systems specifically aimed at rehabilitation. This seems surprising, as these widely used video games are primarily aimed at children and young adults, and do not consider the preferences and requirements of older adults (Laver et al., 2011). Similar results were reported by Lohse et al. (2014), who also found no differences between the effect of commercial video game systems and that of specific rehabilitation systems in terms of functional recovery after stroke. One explanation might be that the samples included in most studies comprised a subgroup of residents with little cognitive and physical impairments. Notably, in one of the few studies with commercial exergames where being wheelchair-bound was not an exclusion criterion, it was reported that there were difficulties with video capturing for residents using electric wheelchairs (Hutchinson et al., 2018).

### 4.6 Strengths and Limitations

This review focuses not only on the effectiveness of e- and m-health interventions, but also integrates acceptability and feasibility into the analysis, providing a broader perspective on the implementation of such interventions. Moreover, in addition to results on PA levels and accidental falls, it includes several other relevant variables regarding the physical, cognitive, and social health of NH residents. Another strength is, that by focusing on NH, it is possible to provide specific recommendations for this setting. Moreover, no restrictions to language or publication date were applied to provide a broad perspective of the literature. Finally, this review includes studies from 17 different countries and six continents and thus does not only reflect western industrialized countries.

However, when interpreting the results of this systematic review, some limitations must be considered. The included studies considerably differed in terms of study design, comorbidities of individuals, intervention characteristics, study duration, and outcomes assessed, which complicated the analysis of results, and a meta-analysis could not be performed. The inclusion and exclusion criteria in many studies on physical and cognitive functioning have ensured a certain homogeneity regarding these two factors. However, since relatively small differences (i.e., able to walk independently vs only able to walk independently with a walking aid) might make large differences, it is critical to note that in the analysis of the results, these factors were not considered in any of the studies (e.g., through the inclusion of a covariate). Additionally, sample size was small in several studies, and few studies reported a rationale for sample size estimation, indicating a risk of limited statistical power in several studies. To obtain a comprehensive overview, non-randomized, non-controlled intervention studies were included. However, this negatively affected study quality, resulting in more than one third of studies being rated as “weak”. Furthermore, it might be difficult to transfer the results to NH residents with severe physical and cognitive impairments. Most studies included only subjects without or with mild cognitive impairment and excluded subjects with severe visual or hearing impairment as well as subjects who could not stand independently or walk a few meters independently with or without walking aid. Additionally, interventions targeting only NH residents with a specific health condition (e.g., only individuals with dementia), robot-assisted interventions and studies that focused exclusively on individuals with a specific health impairment were excluded. Therefore, we cannot provide information on these types of interventions. Another aspect is that there are large differences in NH in terms of quality, service provision, financing, equipment, amenities, and leisure time activities, which vary especially in international comparisons, making it difficult to compare studies (Katz, 2011). Most trials did provide only few information on the setting. Furthermore, different terms like residential aged care or long-term care were used, which were not or not consistently defined.

## 5 Conclusion

The vast majority of studies investigating e-health interventions in NH include exergaming. Remarkably, not a single m-health intervention was identified. Results suggest that exergaming is effective in reducing the number of falls as well as fall risk in NH residents and that it is superior to conventional training in terms of fall risk reduction when the conventional training includes little or no balance training. Based on the included studies, no conclusion can be drawn on the effect of e- and m-health interventions on PA levels in NH residents, as data are very limited. The examined e-health interventions were largely described as feasible and accepted by NH residents. As for secondary outcomes, exergaming resulted in several significant improvements; however, when compared with conventional training, significant differences were rarely found. Remarkably, most studies excluded individuals with advanced cognitive and physical impairments. The proportion of residents for whom commonly used exergames are feasible is difficult to quantify and may depend on various factors. Therefore, other approaches are critically needed which should consider the individual’s physical and cognitive abilities and provide options for individual tailoring. Additionally, the conditions and resources in NH such as equipment and physical space, financial capacities, as well as the knowledge and acceptance of NH staff and residents regarding the respective technology should also be addressed. Practice-oriented research is needed to determine the prevailing environmental conditions to develop digital solutions tailored to these factors. Regarding technology acceptance among NH residents, results suggest that initial concerns about using new technology may diminish after a learning period. This indicates that, to some extent, NH residents are able to engage with new technology. Consequently, they should be provided with support and time to slowly familiarize themselves with a new technology, rather than rejecting the use of such technologies in NH from the beginning. For residents with advanced impairments, m-health applications that empower nursing home staff to support NH residents in achieving sufficient PA levels might be a solution. In view of an increasingly aging population, further studies examining the impact of e- and m-health interventions on PA levels of NH residents are urgently needed.

## Data Availability

The original contributions presented in the study are included in the article/Supplementary Material, further inquiries can be directed to the corresponding author.
