# Supplementary material for: Effectiveness and Acceptability of e- and m-Health Interventions to Promote Physical Activity and Prevent Falls in Nursing Homes—A Systematic Review
Source: Front Physiol. 2022 May 20;13:894397. doi: 10.3389/fphys.2022.894397 (PMC9163679; doi:10.3389/fphys.2022.894397)
Supplement: Supplementary file 1 [file Table1.DOCX]

Supplementary Material

# Supplementary Data

**Systematic reviews on similar topics screened for relevant publications**

Choi, S. D., Guo, L., Kang, D., and Xiong, S. (2017). Exergame technology and interactive interventions for elderly fall prevention: A systematic literature review. *Applied ergonomics* 65, 570–581. doi: 10.1016/j.apergo.2016.10.013.

Fang, Q., Ghanouni, P., Anderson, S. E., Touchett, H., Shirley, R., Fang, F. et al. (2020). Effects of Exergaming on Balance of Healthy Older Adults: A Systematic Review and Meta-analysis of Randomized Controlled Trials. *Games for health journal* 9, 11–23. doi: 10.1089/g4h.2019.0016.

Gauthier-Beaupré, Amélie, Biss, Renée K., Talebzadeh, Arezoo, Sultana, Afroza and Chu (2017). “The effects of exergaming interventions on cognition and physical activity of institutionalized older adults: A systematic review systematic review,” in *9th International Congress of Gerontology: Proceedings*.

Nawaz, A., Skjæret, N., Helbostad, J. L., Vereijken, B., Boulton, E., and Svanaes, D. (2016). Usability and acceptability of balance exergames in older adults: A scoping review. *Health informatics journal* 22, 911–931. doi: 10.1177/1460458215598638.

Oh-Park, M., Doan, T., Dohle, C., Vermiglio-Kohn, V., and Abdou, A. (2021). Technology Utilization in Fall Prevention. *American journal of physical medicine & rehabilitation* 100, 92–99. doi: 10.1097/PHM.0000000000001554.

Pacheco, T. B. F., Medeiros, C. S. P. de, Oliveira, V. H. B. de, Vieira, E. R., and Cavalcanti, F. A. C. de (2020). Effectiveness of exergames for improving mobility and balance in older adults: a systematic review and meta-analysis. *Systematic reviews* 9, 163. doi: 10.1186/s13643-020-01421-7.

Schröder, D. (2021). Exergames zur Förderung der körperlichen Aktivität und Mobilität in der stationären Langzeitpflege – Ein Überblick [Exergames to promote physical activity and mobility in inpatient long-term nursing-An overview]. *Zeitschrift fur Gerontologie und Geriatrie.* doi: 10.1007/s00391-021-01951-2.

# Supplementary Figures and Tables

**Supplementary Table S1**: Search Strategy for each database.

| **Pubmed** | **(ehealth[Title/Abstract] OR e-health[Title/Abstract] OR mhealth[Title/Abstract] OR m-health[Title/Abstract] OR "mobile phone"[Title/Abstract] OR "mobile application"[Title/Abstract] OR telemedicine[MeSH Terms] OR "video gam*"[Title/Abstract] OR "augmented reality"[Title/Abstract] OR "virtual reality"[Title/Abstract] OR wearable*[Title/Abstract] OR "cell phone"[MeSH Terms] OR smartphone*[Title/Abstract] OR "tablet computer*"[Title/Abstract] OR "conversational agent*"[Title/Abstract] OR chatbot*[Title/Abstract] OR Wii[Title/Abstract] OR internet-based[Title/Abstract] OR web-based[Title/Abstract] OR Nintendo[Title/Abstract] OR Xbox[Title/Abstract] OR "Health App*"[Title/Abstract]) AND ("physical activ*"[Title/Abstract] OR activ*[Title/Abstract] OR walk*[Title/Abstract] OR strength[Title/Abstract] OR balance[Title/Abstract] OR exercise[MeSH Terms] OR function*[Title/Abstract] OR mobility[Title/Abstract] OR train*[Title/Abstract] OR gait[MeSH Terms] OR fall*[Title/Abstract]) AND ("Nursing Homes"[MeSH Terms] OR "Nursing Home*"[Title/Abstract] OR "Long-Term Care"[Title/Abstract] OR "Long Term Care"[Title/Abstract] OR "Residential Facilities"[Mesh:NoExp] OR "Residential Facilit*"[Title/Abstract] OR "Homes for the Aged"[MeSH Terms] OR "Care home* resident*"[Title/Abstract] OR "Retirement Home*"[Title/Abstract] OR Institutionali?ed[Title/Abstract] OR "aged care facilit*"[Title/Abstract]) Filters: Journal Article** |
| --- | --- |
| **Scopus** | TITLE-ABS-KEY ( ehealth OR e-health OR mhealth OR m-health OR "mobile phone" OR "mobile application" OR telemedicine OR "video gam*" OR "augmented reality" OR "virtual reality" OR wearable* OR "cell phone*" OR smartphone* OR "tablet computer*" OR "conversational agent*" OR chatbot* OR wii OR internet-based OR web-based OR nintendo OR xbox OR "Health App*" ) **AND** TITLE-ABS-KEY ( "physical activ*" OR activ* OR walk* OR strength OR balance OR exercis* OR function* OR mobility OR train* OR gait OR fall* ) **AND** TITLE-ABS-KEY ( "nursing home*" OR "Long-Term Care" OR "Long Term Care" OR "Residential Facilit*" OR "Home* for the Aged" OR "Care home* resident*" OR "Retirement Home*" OR institutionali?ed OR "aged care facilit*" ) **AND** ( LIMIT-TO ( DOCTYPE , "ar" ) ) |
| **SPORTDiscuss** | ehealth OR e-health OR mhealth OR m-health OR "mobile phone" OR "mobile application" OR telemedicine OR "video gam*" OR "augmented reality" OR "virtual reality" OR wearable* OR "cell phone*" OR smartphone* OR "tablet computer*" OR "conversational agent*" OR chatbot* OR Wii OR internet-based OR web-based OR Nintendo OR Xbox OR "Health App*" **AND** "physical activ*" OR activ* OR walk* OR strength OR balance OR Exercis* OR function* OR mobility OR train* OR gait OR fall* **AND** "nursing home*" OR "Long-Term Care" OR "Long Term Care" OR "Residential Facilit*" OR "Home* for the Aged" OR "Care home* resident*" OR **"Retirement Home*" OR Institutionali?ed** OR **"aged care facilit*" Filter: Academic Journals** |
| **Web of science** | (TS=(ehealth) OR TS=(e-health) OR TS=(mhealth) OR TS=(m-health) OR TS=("mobile phone") OR TS=("mobile application") OR TS=(telemedicine) OR TS=("video gam*") OR TS=("augmented reality") OR TS=("virtual reality") OR TS=(wearable*) OR TS=("cell phone*") OR TS=(smartphone*) OR TS=("tablet computer*") OR TS=("conversational agent*") OR TS=(chatbot*) OR TS=(Wii) OR TS=(internet-based) OR TS=(web-based) OR TS=(Nintendo) OR TS=(Xbox) OR TS=("Health App*")) **AND** (TS=("physical activ*") OR TS=(activ*) OR TS=(walk*) OR TS=(strength) OR TS=(balance) OR TS=(Exercis*) OR TS=(function*) OR TS=(mobility) OR TS=(train*) OR TS=(gait) OR TS=(fall*)) **AND** (TS=("nursing home*") OR TS=("Long-Term Care") OR TS=("Long Term Care") OR TS=("Residential Facilit*") OR TS=("Home* for the Aged") OR TS=("Care home* resident*") OR TS=("Retirement Home*") OR TS=(Institutionali?ed) OR TS=("aged care facilit*")) **Filter**: Document Types: Articles |
| **Google scholar (Additional search)** | Exergaming OR ehealth OR mHealth OR "mobile phone" **AND** "physical activity" OR balance OR fall **AND** "nursing home" OR "Long-Term Care" OR "Long Term Care" OR "Residential Facility" OR institutionalized |
